# Treatment plan for breast cancer with sentinel node metastasis

**DOI:** 10.3332/ecancer.2014.383

**Published:** 2014-01-08

**Authors:** Efrén Bolívar Abreu, Pedro Martinez, Luis Betancourt, Gabriel Romero, Ali Godoy, Laura Bergamo

**Affiliations:** Breast Pathology Department, Dr Luis Razetti Oncology Institute, Caracas, Venezuela

**Keywords:** breast cancer, sentinel node, macrometastases, micrometastases

## Abstract

Lymph node involvement is considered to be one of the most important independent prognostic factors in breast cancer. In patients without palpable lymphadenopathies, the method of choice for determining this involvement is the sentinel lymph node biopsy. In the presence of macrometastases, the current standard is to perform axillary lymph node dissection in spite of the knowledge that the involvement of non-sentinel lymph nodes is approximately 50%. When lymph node involvement is micrometastasic, the decision as to whether or not to proceed with lymphadenectomy remains in dispute. We set out, on the basis of the current scientific evidence and our own experience, to create guidelines that allow us to individualise each case and decide whether or not to perform a lymphadenectomy. We will discuss the arguments that support our position.

## Introduction

Axillary involvement is considered to be the most important independent prognostic factor in breast cancer. In the last 15 years, the number of patients with breast cancer benefiting from sentinel lymph node biopsy (SLNB) has increased [1, 2]. When the sentinel node is affected, axillary lymphadenectomy has been the procedure of choice. This is based on the fact that it provides key information for the adjuvant therapy, serves as a prognostic element and improves local control [3]. The possibility of additional metastatic disease in the axilla when the SNB is positive is between approximately 40% and 50%. Therefore, in this situation, current accepted practice continues to be axillary lymph node dissection (ALND) [4]. In contrast, a significant group of patients do not have any additional axillary disease and if we take into account morbidity and operative time and costs, skipping the procedure would be beneficial to these patients. For this reason, one way to address this problem is to relate the known clinicopathological factors in breast cancer, with the probability of residual disease in the axilla, allowing for a selection of cases in which ALND would not be performed [5].

There are other elements that explain the growing interest in omitting ALND. The majority of patients with SLN involvement will receive adjuvant chemotherapy, and its indications do not depend on the number of lymph nodes with metastasis. In contrast, for a significant group of patients, the decision making in adjuvant treatment planning will be based on the immunohistochemical study of the tumour, which has a greater influence in the therapeutic decision than the ALND. Finally, during the years 2010 and 2011, the first results of clinical trials were published that specifically analysed the impact of ALND on local control and survival of women with SLN involvement. These have not shown any benefit in patients who had an ALND performed [6]. In light of these facts, the balance has begun to lean towards a change in attitude towards a positive SLN justifying ALND only in selected cases.

## Scientific evidence

The ACOSOG Z0011 multicentre clinical trial by Giuliano *et al* [7] shows that women with early-stage breast cancer and sentinel node metastases do not benefit from ALND. Therefore, they have come to the conclusion that there are no changes in overall survival or diseasefree survival at five years or in local recurrence [8]. The criteria for inclusion in the study were patients with a diagnosis of breast carcinoma T1/T2-N0-M0, treated with breast-conserving surgery with one or two positive SLN. Metastases diagnosed by immunohistochemistry were excluded, as well as patients with more than two positive SLN, lymph node adhesions or specific indications for radiation therapy to the axilla. In total, there were 891 patients, 445 had ALND and 446 did not receive additional post-surgical treatment after the SLNB. The average time of follow-up was 6.3 years. All patients received radiation therapy in two tangential fields.

In the analysis of the results, it is noted that age (young patients) and small degree of differentiation were related to a greater percentage of locoregional relapse. Non-sentinel lymph node involvement was 27%, a fact observed in the arms of patients undergoing ALND, suggesting that in the group of patients under observation there was a similar trend. However, since there were no differences in relapse, the importance of the adjuvant treatments is highlighted as elements of locoregional control, suggesting that ALND does not improve this control.

However, it is important to take into account the limitations of this work. The main being that the selection of patients was mainly among those in very early-stage cases with good prognostic factors (70% with stage T1, 83% with oestrogen receptor positive, 35% with SLN micrometastases in the arm and 44.8 % with micrometastasic disease in the non-ALND group). In addition, in all cases, radiation therapy to the entire breast bed was administered, which influences the possible extrapolation to less extensive radiotherapy fields. There was limited follow-up; 6.3 years being the median time. Ninety-six per cent of patients received chemotherapy. Recruitment of the expected number of patients (891 of 1900 planned) was not possible for reasons related to patients themselves or to the denial of some surgeons. Eighty percent of patients were hormone receptor positive, which skews the sample towards tumours with better prognosis, susceptible to hormonal manipulation. There was no identification of Her-2/neu at the start of the study as this was not a standard procedure. This limits the establishment of subgroups of patients, including the triple negative [6, 8].

There are other studies that have looked into sentinel node involvement. However, the majority have focused on the analysis of micrometastases. One of these is the MIRROR study, in which nearly 2600 patients with biologically favourable tumours, and no indication for systemic adjuvant treatment with chemotherapy, participated in a trial. These patients had negative sentinel lymph nodes PN0, isolated tumour cells pN0 (i+), or were positive for micrometastases pN1mi, and were separated randomly into ALND or axilla radiation therapy. In this study, omission of axillary dissection or radiotherapy in patients with a positive SLNB for micrometastases resulted in a higher rate of axillary relapse at five years [9].

Galimberti *et al* conducted a study in the European Institute of Oncology in which they omitted ALND in 377 patients with micrometastases in SLN; 2.1% (*N* = 8) of the patients had a recurrence at the axillary level. The only risk factor detected for axillary relapse was the size of the tumour [10].

In the NSABP-B32 trial, analysing the clinicopathological factors associated with positive non-sentinel lymph nodes was one of the objectives of the study. The tumour size, presence of lymphovascular invasion, and SLN macrometastases were independent predictors. There was no cutoff point considered for the size of the tumour since this was considered to be a continuous variable (greater size, greater involvement) [11].

In a study conducted in Barcelona, Spain, by Pernas and colleagues, 59 cases with SLN micrometastasic involvement in which ALND was not performed were analysed. There were no axillary recurrences in a median follow-up of 60 months [12]. In another similar study carried out by Langer and colleagues, 27 patients with SLN micrometastases who were not subjected to ALND had no relapse in 42 months of follow-up [13].

Finally, in April of this year, the results of the IBCSG Trial 23-01 were published. This trial was conducted in 27 European centres in which 931 women with SLN micrometastases involvement were randomly assigned to ALND or not. The majority (85%) had one or two positive SNL. With a median follow-up of 57 months, the disease-free survival rate was 87.3% and 88.4% (*P* = 0.48), with an overall survival of 97.6% and 98% (*P* = 0.35) for the groups assigned ALND and not, respectively [14].

We can see that there are differing results between the various studies, but that in general, all results suggest that there are safe options (observation, radiation therapy, systemic treatment) that arise as alternatives to ALND in a subgroup of patients with SLN involvement.

### Experience of the Dr Luis Razetti Oncology Institute

In our Breast Pathology Department, we are continuously searching for solid foundations for our medical practice that ultimately translate into benefit for patients. SLN involvement has been one of these fields of research. In 2011, a study was published by Zenzola *et al* [5] in which the main objective was to analyse clinicopathological factors in patients with positive SLNB, to be able to predict the non-sentinel lymph node involvement and in this way bypass ALND in selected patients. In our study, three factors were statistically significant in the univariate analysis to predict the status of non-sentinel lymph nodes: nuclear grade III, the presence of lymphovascular invasion, and the number of positive sentinel lymph nodes (two or more). Only the presence of lymphovascular invasion was an independent predictor (multivariate analysis) of metastases in non-sentinel lymph nodes. It was concluded that the presence of lymphovascular invasion significantly increases the possibility of residual disease after a positive axillary sentinel node biopsy. The presence of additional disease after a positive biopsy is considerable (50%).

## Thesis

### Radiotherapy

Given the possibility of residual disease in the underarm when the SLN is positive in patients selected for skipping ALND, most centres tend to prescribe radiation treatment at that level as a tool of local control. In fact, the incidence of axillary relapses in women with SLN (micrometastasis or macrometastasis) without ALND is the same as in women with ALND when combined with postoperative radiotherapy in the breast, and this is based on prospective randomised clinical trials [15, 16]. Patients undergoing breast-conserving surgery receive radiotherapy as part of the integral treatment. Tangential fields affect the underarm in a variable way depending on the planning, mainly at Berg node levels I and II [17]. However, radiotherapy must be undertaken where axillary irradiation is guaranteed with a therapeutic dose in diseases where ALND is not done in order to improve local control. In a trial in the European Oncology Institute by Veronesi *et al*, clinically negative node (cN0) patients with tumours up to 1.2 cm were randomised to receive either axillary radiotherapy or no axillary radiotherapy. With an average follow-up of 63 months, no differences were found between the working underarms in terms of local recurrences and disease-free survival [18]. However, more information about this is needed, such as from the phase III study EORTC 10981-22023 After Mapping of the Axilla: Radiotherapy Or Surgery (AMAROS) trial, which is currently done by randomising patients with positive SLN in two working underarms: ALND or axillary irradiation. In a preliminary report (.) of this trial, both groups were observed that had survival rates comparable with the double burden of disease in terms of lymphoedema in the underarm of the axillary lymphatic dissection [19].

### Use of predictive models

At least nine mathematical models have been developed to predict the status of non-sentinel lymph nodes in patients with breast cancer and SLN. Among them are included four nomograms (Memorial Sloan-Kettering Cancer Center (MSKCC), Mayo, Cambridge, Stanford), three scoring systems (Tenon, MD Anderson, Saidi), and two recursive partitioning models (Kohrt *et al*). Through these methods, attempts are made to establish quantitatively the probability of metastasis in non-sentinel lymph nodes, taking into account factors like tumour size, size of the metastasis in the SLN, number of positive SLNs, ratio between the number of positive SLNs and removed SLNs, lymphovascular invasion, extracapsular extension, among others [20]. The model distributed most in clinical practice and which has also been validated by several authors is the nomogram of MSKCC. In it, it takes into account nine clinical and pathological variables, presenting the most discrimination to predict non-sentinel lymph node diseases [21]. These methods are useful tools that help select patients for either ALND or no ALND. However, many lose their applicability when they are used outside of the institution of origin for probable differences between patients of different latitudes and also have the disadvantage of not incorporating elements like the Her2/neu status, molecular subtypes or genetic signatures, which are getting more and more important at the time of therapeutic decision-making in current medical practice. Let us consider that they may be of use in different working groups that, coupled with other factors and never as a single element, serve as a tool in decision-making.

### Importance of molecular subtypes

Perou *et al* established molecular subtypes of breast cancer [22], which has acquired greater relevance at the time of establishing the prognosis and therapy for patients. Genetic signatures, which allow for identifying said subtypes, also allow for the establishment of the probability of recurrence and the need for chemotherapy. There is a high level of concordance between the molecular subtypes and the expression of either phenotypic elements or no phenotypic elements (hormonal receptors, Her2/neu, KI 67), which allows close characterisation through immunohistochemistry in places where this type of tools are not used (Oncotype, Mammaprint).

Upon establishing the molecular and phenotypic characteristics of the tumour, it is possible to set up an individualised treatment, directed to this specific subtype, with which better control of the disease is ensured. Therefore, let us consider of vital importance its use in therapeutic decision-making, both medical and surgical. One example is the triple-negative phenotype, which constitutes a group of worse prognoses with a high regional and distant metastatic potential, where the single systemic treatment to use is chemotherapy and which therefore must be treated from the most appropriate surgical point of view. Other subtypes are of a better prognosis and are susceptible to more alternatives in treatment.

### Intraoperative outcome of the SLNB

Standard medical practice during the SLN biopsy is to wait for the outcome of the intraoperative biopsy (frozen section), and in case, the report is positive for metastasis, to proceed to ALND. This avoids the possibility of a second surgical intervention since the procedure is completed in just one stage. However, we see that there is a series of clinical and pathological factors, some of the primary tumour (tumour size, lymphovascular invasion, etc.) and others of the SLN (size of the metastasis, extracapsular invasion, etc.) that are important for decision-making and that they are not considered but after the definitive anatomopathological study. This raises the question of whether to conduct a preoperative histological study of the SLN or to wait on a more exhaustive study to obtain more complete information and make the most appropriate determination. In both MD Anderson and MSKCC, as current everyday practice, they have the omission of the intraoperative study of the SLN, in patients who meet the criteria of the ACSOG Z0011, while waiting on the outcome of the definitive biopsy for decision-making [23, 24].

## Guidelines for decision-making

The Venezuelan Mastology Society published its Consensus of Sentinel Lymph Node in Mammary Carcinoma in 2010. This recommends performing ALND in all cases of macrometastasis and leaves the decision in the hands of each working group in cases of micrometastasis. The American Society of Clinical Oncology (ASCO) recommends performing ALND in all patients with SLN, whether they have macro or micrometastasis.

In our Mammary Pathology Service, we conduct a presurgical discussion, individualising the decision for each case. We propose on the basis of existing evidence and our own evidence, the following criteria for omitting ALND, in cN0 patients:

1. Tumours under 2 cm (T1).
2. One or two affected sentinel lymph nodes.
3. Absence of infovascular invasion.
4. Guaranteed irradiation of Berg levels I and II.
5. Guaranteed adjuvant systemic treatment.
6. Positive hormonal receptors.

The absence of just one of the criteria indicates performing ALND.

We recommend waiting for the outcome of definitive pathologic anatomy in cases where no sufficient elements are considered for deciding whether or not to perform ALND. We suggest not omitting ALND in cases of triple-negative tumours or overexpressed Her2/neu carcinoma that cannot receive treatment with trastuzumab. We also recommend performing axillary dissection in cases of patients who undergo total mastectomy (since the majority will not receive radiotherapy).

## Conclusion

These are the guidelines of the Mammary Pathology Service of the Dr Luis Razetti Oncology Institute for establishing therapeutic conducts in patients with mammary carcinoma and sentinel lymph nodes metastasis. They are based on the experience of our institution, on our own scientific evidence and on other global centres. Medicine is dynamic and these will continue to evolve as they continue to generate new evidence. We hope these guidelines are able to serve as a guide to other working groups.
